# Exploring the association between emergency hospital services and homicide incidents in Pennsylvania

**DOI:** 10.1016/j.ssmph.2024.101744

**Published:** 2024-12-28

**Authors:** Mingean Park, Sujeong Park, Youngeun Lee, Jonathan Lee

**Affiliations:** a School of Public Affairs, Penn State Harrisburg, 777 W. Harrisburg Pike, Middletown, PA, 17057, USA; b, School of Public Health, University of Maryland, College Park, 4200 Valley Dr, College Park, MD, 20742, USA

**Keywords:** Health services, Emergency hospital, Homicide, Pennsylvania, Spatial analysis

## Abstract

Homicide is a significant measure of quality of life and serves as a reference point for a comparison between neighborhoods. Despite its unique relevance to homicide, the role of medical resources, specifically emergency hospital services, has been underexplored in the literature. This study addresses this gap by examining the relationship between emergency hospital availability and homicide rates across counties in Pennsylvania, using advanced spatiotemporal modeling techniques. While controlling for socio-economic characteristics and spatial autocorrelation, the analysis suggests that greater access to emergency hospital services is associated with lower homicide rates. These findings call for the importance of medical resources in both future homicide research and health policy.

## Highlights

• Homicide rates from 2013 to 2019 across counties in Pennsylvania were examined for their association with emergency hospitals.
• Counties with more emergency hospitals showed a lower homicide rate. while controlling for socio-economic factors and spatial contiguity of homicides.

## 1 Introduction

A crime rate measures the prevalence of violence in a given space and time and serves as a barometer of public wellbeing. Criminological theories have discussed how geographic concentration of crime depends on sociological factors and the physical environment of a region. Social disorganization theory of crime and delinquency discusses the socio-economic characteristics of a high-risk neighborhood such as residential mobility, concentrated disadvantage, and racial heterogeneity (Sampson & Groves, 1989). Empirical studies have measured those characteristics using education, poverty and unemployment rates, female-headed household rate, and property occupancy rate, among others (Ahlfeldt & Pietrostefani, 2019; Boessen & Chamberlain, 2017; Boessen & Hipp, 2015; Branas et al., 2012; Cui & Walsh, 2015; Dobrin et al., 2005; Hipp & Yates, 2011; Lanier, 2010; Lee et al., 2016; Lichter, 2012; Lochner & Moretti, 2004; Meng, 2021; Nordin & Almén, 2017; Phillips & Land, 2012; Pridemore, 2011; Raleigh & Galster, 2015; Sameem & Sylwester, 2018; Tur-Prats, 2021). Similarly, the rational choice perspective in criminology addresses how a specific set of spatial and temporal environments constitutes an optimal condition for crime (Lee et al., 2013). As criminogenic establishments are geographically clustered, for example, crimes would follow a similar pattern, such as 80-20 rule that 20% of a city area account for 80% of all crimes (Clarke & Eck, 2005). The literature identified bars, alcohol outlets, vacant buildings, and theme parks as common geospatial characteristics of crime hot spots (Ashe et al., 2003; Caplan et al., 2021; Gorman et al., 2001; Han et al., 2021; McCord & Ratcliffe, 2009; Stacy, 2018). Health behaviors, such as alcohol consumption and smoking behavior, have also been examined as factors of crime (Darke et al., 2013; Lester, 1995; van Breen & Liem, 2024). For example, Parker et al. (2011) found a positive association between youth homicide and alcohol availability using data from the U.S. Department of Justice's Supplemental Homicide Report among 91 cities from 1984 to 2006.

Homicide, among others, has been the most popular measure of violence for a few methodological advantages. It is defined as the death of a person that is not due to a natural cause or self-inflicted harm. Because it has a relatively simple definition and no other higher category, it is less prone to confusion or definitional idiosyncrasies across jurisdictions than other types of crime. For example, possession of marijuana may be a crime in one state while not in another. Theft of merchandise worth $1000 would be a felony in Ohio while it would be a misdemeanor in Maryland as of 2016. In addition, a single criminal case often involves multiple offenses at varying degrees of seriousness. In order to minimize duplicity in the records, the FBI counts only the most serious offense in each case. For example, if a theft escalates into a robbery, which in turn results in the death of a victim, the case would be counted as a homicide, undercounting a theft and a robbery for the jurisdiction. For the reasons above, a number of empirical research studies have examined homicide rate as either a predictor or an outcome variable across neighborhoods (Becker, 2020; Daly et al., 2001; Jones-Webb & Wall, 2008; Kubrin & Herting, 2003; Menezes et al., 2013; Siegel et al., 2013; Thompson & Gartner, 2014).

Homicide is unique in that, unlike other offenses, its definition depends on the status of the victim's life. For instance, a stabbing or even a gunshot may not instantly claim the life of a victim. If the victim survived, the case would not count as a homicide. Such a distinctive aspect of homicide warrants a broader scope of determinants than for most other offense types. While likely correlate with gun violence, for example, criminogenic factors such as high population density in the neighborhood would not contribute to the odds of a victim's survival from a gunshot wound. In other words, a homicide rate depends on not only the factors of violence but also the factors of the victim's chance of survival, such as emergency medical services.

Medical services in the U.S. have grown in their quality and quantity over the last several decades. Acute pains and serious bodily injuries are the areas where the advancements stand out because the results are measurable through patient reports and the mortality rate. It is particularly so for gunshot wounds because advanced medical services can save lives that would have been lost had it not been for the advancements (Toussaint & Gerard, 2010). In addition to the quality of services, the availability of those services has grown over the years in the U.S., which translates to greater accessibility to urgent medical care (Finks et al., 2011; Tang et al., 2010).

Albeit limited, the relationship between medical resources and crime, particularly homicides, has been addressed in the literature. As Barlow and Barlow (1988) noted, survival chances drastically increase if medical treatment is received within 20 min of an attack. Summers and Rogers (2020) found that spatial proximity to emergency care providers can mitigate homicide lethality, while Harris et al. (2002) argue that medical advances should be credited for the homicide rate drop in the U.S. between 1960 and 1990. Chon (2010)'s cross-national study reports the inverse association between the number of nurses and the homicide rates. Doerner and Speir (1986) studied 67 counties in Florida, highlighting the importance of emergency medical resources, such as the number of hospital beds and physicians, in relation to crime rates. Other empirical studies have reported that a lower homicide rate could be attributed to more advanced as well as more available medical services at various levels of aggregation (Aebi & Linde, 2010; Long-Onnen & Cheatwood, 1992). Analogous findings were reported from European studies as well. Estrada (2006) examined long-term trends in violent crimes in Sweden from 1974 to 2002 and found that the improvements in healthcare practices may have contributed to an increase in victims' survival rates from violence. Similarly, Linde (2017) found that healthcare improvements have contributed to a decrease in homicide victims in Germany.

The current study hypothesized that a homicide rate hinges on medical resource accessibility. It also aims to advance the literature by employing a spatial econometrics model. Spatial interdependence occurs when a geographic unit is influenced by the conditions of nearby units, creating non-random spatial patterns (Anselin, 1988; Cook et al., 2020). For example, high unemployment in one city increases job demand in nearby cities (LeSage & Pace, 2009), and pollution can spread across regional borders (Levinson, 2009). When sociological factors and physical environment are associated with crime, geographical contiguity of those factors between two adjacent areas warrants a strong likelihood of comparable crime rates between the two. Consistent with mimetic isomorphism (DiMaggio & Powell, 1983), high-crime neighborhoods tend to cluster because adjacent neighborhoods share similar socio-environmental conditions due in no small part to the reciprocal dependency between crime and environment (Cook et al., 2019, 2023). It resonates with spatial autocorrelation of crime as crimes in one area beget crimes in nearby area. Quantitative measures like Moran's *I* have been introduced to assess the presence and strength of spatial contiguity, specifically measuring spatial autocorrelation to estimate the extent to which a variable is similarly distributed across geographic units (Li et al., 2007; Moran, 1950). However, prior studies on homicide have not considered spatial autocorrelation of homicide or spatial effect of neighborhood conditions as a standalone factor (Summers & Rogers, 2020). Our study seeks to fill this gap by accounting for the effect of both homicides and hospitals in neighboring areas on the homicides in an area.

## 2 Data and methods

### 2.1 Sample/variables

The sample for the current study consists of 67 Pennsylvania counties. All variables used in this study are panel data measured at the county-year level, ranging from 2013 to 2019. The time frame was chosen to capture recent trends while ensuring data availability and consistency and to exclude the potential effects of the COVID-19 pandemic. The data consists of 67 counties from 2013 to 2019, resulting in a total sample of 469 observations (67 counties × 7 years).

The outcome variable is the homicide rate per 100,000 people, presented as a standardized measure across counties of varying population (see Table 1). The data were retrieved the yearly county-level homicide counts between 2013 and 2019 from the *Offenses Known and Clearances by Arrest (Return A), 1960*–*2020* dataset provided by the Inter-university Consortium for Political and Social Research (Kaplan, 2021). As a measure of emergency medical services availability, the number of emergency hospitals for each county was acquired from *Hospital Reports* data from the Pennsylvania Department of Health (Pennsylvania Department of Health, 2022). The number was standardized as the count of emergency hospitals per acre in order to control for the varying sizes of area across counties.

**Table 1:** Descriptive statistics.

Variable	Obs.	Mean	Std. Dev.	Min	Max
Homicide Rate (per 100,000)	469	0.202	0.190	0.000	1.860
Emergency Hospitals (per acre)	469	5.264	14.468	0.000	126.771
Health Index	469	12.947	3.460	5.700	26.000
Racial Diversity Index	469	0.174	0.131	0.040	0.660
% Female-head Householder	469	0.222	0.050	0.140	0.480
Occupancy Rate	469	0.845	0.125	0.290	0.950

Also used in the current study is a health index which encompasses aforementioned criminogenic factors such as health behaviors, social and economic factors, and the physical environment (see Table 1). The index is a part of the health factors in the *County Health Rankings*, and has been published annually by the University of Wisconsin Population Health Institute since 2010 (Remington et al., 2015). Additional criminogenic factors that are well-documented in the literature and observable at the county level were included in the current study. The racial diversity index is calculated using the proportions of racial groups in each county (Blau, 1977; Gibbs, Lee, Moloney, & Olson, 2018). The index estimates the probability of two random individuals in the county being of different races. The female-headed household rate is the percentage of households with a female head among the families with one or more children under 18 years old. The occupancy rate measures the percentage of housing units that are occupied as opposed to vacant in a county. These statistics were retrieved from the American Community Survey data for the years 2013 through 2019 (U.S. Census Bureau, 2024).

### 2.2 Models

To ensure the robustness of our findings, we employed four different modeling techniques, including ordinary least squares (OLS), OLS with fixed effects, the Spatial Autoregressive Model (SAR), and the Spatial Durbin Model (SDM). Our dependent variable, the homicide rate, is measured as the number of victims per 100,000 population, making it a continuous variable. Therefore, count-variable models such as negative binomial or Poisson are not applicable in this study (Wooldridge, 2015). We also confirmed that the goodness-of-fit for both negative binomial and Poisson regression was relatively deficient when compared to OLS estimation model. The use of fixed effects in the OLS model was particularly useful in controlling for unobserved, time-invariant differences across counties, helping to reduce bias from factors that vary between counties but remain constant over time. In addition, spatial models (SAR and SDM) allowed us to capture both the ceteris paribus effect of socio-environmental variables and spatial dependence between counties, ensuring consistency across various analytical frameworks (Anselin, 1988; Cook et al., 2019, 2023).

Spatial autocorrelation should be confirmed before executing the spatial model. Our spatial analysis found that Moran's *I* was 0.442, indicating the presence of spatial autocorrelation in the homicide rate across Pennsylvania counties in 2019. Using Pennsylvania county-level spatial autocorrelation information, a spatial weighting matrix was created for analytic models. The queen method of assigning through contiguity to reflect spatial interaction among counties was adopted, and $\rho \;W_{i}^{'}$ was acquired by regressing the homicide rate against the spatial weighting matrix.

(1) $$w=\left[\begin{array}{ccc} w_{1\;1} & \cdots & w_{67\;j}\\ \vdots & \ddots & \vdots \\ w_{i\;67} & \cdots & w_{67\;67} \end{array}\right]$$

(2) $$\text{Normalization}\;\text{of}\;i\;\text{county}’s\;w,1=\sum_{j=1}^{67}w_{ij}$$

(3) $$i\;\text{county}’s\;w=\left[w_{1}w_{2}\cdots w_{67}\right]$$

Model 1 is a conventional OLS for the association between emergency medical services and the homicide rate. Model 2 includes county fixed-effect terms to control for county-level heterogeneity (Wooldridge, 2015).

Model 3 is the SAR that includes the spatial lag term ($W_{i}^{\prime }$**),** measures the influence of neighboring counties' homicide rates on each county's homicide rate. This model helps capture the spillover/diffusion effects of homicide rates from neighboring counties (Cook et al., 2019). Model 4 is the SDM that includes the spatial lag terms of both the dependent variable (homicide rate; $\rho W_{i}^{\prime }y$) and the independent variable (emergency medical services; $W_{i}^{\prime }X\theta$). This model allows for a stricter interpretation of the regional interdependency among counties by considering how both crime rates and medical services in neighboring counties influence a county's homicide rate (Cook et al., 2019, 2023). All spatial models included county fixed-effect to control for unobserved heterogeneity across counties in the Pennsylvania (Wooldridge, 2015). The variance inflation factor for all variables was checked to dismiss a concern for multicollinearity (Wooldridge, 2015).

$$\left[\;\text{Model}\;1\right]:Crime_{it}=\beta_{0}+\beta_{1}EM_{it}+\beta_{n}ControlVariables_{it}+\varepsilon_{it}$$

$$\left[\;\text{Model}\;2\right]:Crime_{it}=\beta_{0}+\beta_{1}EM_{it}+\beta_{n}ControlVariables_{it}+\delta_{i}+\varepsilon_{it}$$

$$\left[\;\text{Model}\;3\right]:Crime_{it}=\beta_{0}+\rho W_{i}^{\prime }Crime_{t}+\beta_{1}EM_{it}+\beta_{n}ControlVariables_{it}+\delta_{i}+\varepsilon_{it}$$

$$\left[\;\text{Model}\;4\right]:Crime_{it}=\beta_{0}+\rho W_{i}^{\prime }Crime_{t}+\beta_{1}EM_{it}+W_{i}^{\prime }EM_{t}\theta +\beta_{n}ControlVariables_{it}+\delta_{i}+\varepsilon_{it}$$

Note: δ = fixed effect; i = counties; t = year; ε = error term

## 3 Results

The findings from the regular OLS model, reported in Column 1 in Table 2, show that a county with more emergency hospitals per acre (0.005) experienced a higher homicide rate. Concerning the control variables, the health index (0.408), the racial diversity index (0.281), and female-head householder (1.271) are positively associated with the homicide rate.

**Table 2:** Emergency hospital and homicide rate.

	(1)	(2)	(3)	(4)
	OLS	FE	SAR-FE	SDB-FE
Spatial Lag ($\rho W_{i}^{\prime }y$) of Homicide Rate			0.338∗∗∗	0.556∗∗∗
			(0.031)	(0.033)

Spatial Lag ($W_{i}^{\prime }X\theta$) of Emergency Hospitals				−0.006∗∗∗
				(0.001)

Emergency Hospitals	0.005∗∗∗	−0.032∗∗∗	−0.030∗∗∗	−0.029∗∗∗
	(0.001)	(0.003)	(0.002)	(0.002)

Health Index	0.102∗∗∗	−0.057	−0.062∗	−0.059∗
	(0.019)	(0.032)	(0.028)	(0.024)

Racial Diversity Index	0.408∗∗∗	1.277∗∗	1.490∗∗∗	1.280∗∗∗
	(0.055)	(0.398)	(0.350)	(0.307)

Female-head Household rate	0.556∗∗	3.450∗∗∗	2.747∗∗	2.706∗∗∗
	(0.197)	(0.986)	(0.867)	(0.759)

Occupancy rate	−0.068	−3.141∗∗∗	−3.673∗∗∗	−3.394∗∗∗
	(0.053)	(0.831)	(0.730)	(0.640)

Constant	0.038	2.241∗∗	2.759∗∗∗	2.527∗∗∗
	(0.045)	(0.748)	(0.658)	(0.577)

Observations	469	469	469	469
R-squared	0.643	0.871	0.901	0.924
AIC	−699.361	−1049.14	−1171.57	−1295.01
BIC	−674.457	−762.745	−881.028	−1000.32

Note: standard errors in parentheses. ∗p < 0.05, ∗∗p < 0.01, ∗∗∗p < 0.001. VIF (Emergency Hospitals = 2.19; Health Index = 2.73; Racial Diversity Index = 1.85; Female-head Household Rate = 3.49; Occupancy Rate = 1.55).

Column 2 presents the fixed effect estimates, accounting for county-fixed effects. An increase in emergency hospitals per acre (−0.032) is found to have a negative correlation with the homicide rate. Moreover, increases in the occupancy rate (−3.141) also show negative correlations with the homicide rate. In contrast, increases in the racial diversity index (1.277) and female-headed households (3.450) are positively correlated with the homicide rate.

Column 3 indicates the result of the spatial model, which includes the spatial lag of the dependent variable ($\rho W_{i}^{\prime }y$), the homicide rate. This indicates that a county's homicide rate is positively influenced by the homicide rates of neighboring counties. Specifically, a 1-unit increase in the homicide rate of adjacent counties is associated with approximately a 0.338 increase in the homicide rate of the given county. The increase in the key independent variable, emergency hospitals per acre (−0.030), is associated with a negative correlation with the homicide rate. Furthermore, increases in both the health index (−0.062) and the occupancy rate (−3.673) demonstrate a negative relationship with the homicide rate. Conversely, higher values of the racial diversity index (1.490) and female-headed household rate (2.747) correspond to a positive correlation with the homicide rate.

Column 4 displays the results that include both the spatial lags of the homicide rate ($\rho W_{i}^{\prime }y$) and emergency hospitals per acre ($W_{i}^{\prime }X\theta$). The spatial lag of the homicide rate ($\rho W_{i}^{\prime }y$) suggests that a county's homicide rate is affected by the homicide rates of its neighboring counties. Specifically, when the homicide rate in adjacent areas increases by 1-unit, the homicide rate in area county rises by 0.556. The increase in the main independent variable, emergency hospitals per acre (−0.029), is associated with a negative correlation with the homicide rate. Next, the spatial lag of emergency hospitals per acre ($W_{i}^{\prime }X\theta$) shows that an increase of 1-unit of emergency hospitals per acre in neighboring counties is associated with a decrease of 0.006 in the homicide rate in a county. Altogether, a county's homicide rate is associated with both the homicide rates and emergency hospitals in neighboring counties, as well as its own emergency hospitals. The control variables - the health index (−0.059) and the occupancy rate (−3.394) - exhibit negative correlations with the homicide rate. In contrast, rises in the racial diversity index (1.280) and the female-headed household rate (2.706) are positively correlated with the homicide rate.

## 4 Discussion and conclusion

This study builds on the unique understanding that regional variations in homicide rates are influenced not only by socio-economic factors but also by factors related to the likelihood of a victim's survival. The primary objective of this study was to investigate the relationship between emergency hospitals and homicide rates in Pennsylvania. The findings suggest that a higher number of emergency hospitals is associated with a lower homicide rate. The increased availability of emergency hospitals is likely to improve access to trauma care, which may indirectly enhance the promptness of medical intervention. This notion aligns with prior studies suggesting that timely access to emergency care can increase survival rates in violent incidents (Summers & Rogers, 2020). Also, hospitals at nearby counties appeared to lower the homicide rate in a county. Counties surrounded by high-homicide areas tend to experience a high crime rate, suggesting the presence of spatial autocorrelation. The control variables, such as racial diversity, and female-headed households, were consistent with the literature (Dobrin et al., 2005; Hipp & Yates, 2011; Lanier, 2010; Lin, 2008; Phillips & Land, 2012; Pridemore, 2008, 2011), which highlight these factors as contributors to higher homicide rates.

However, the findings should be interpreted with caution. First, the scope of the study was limited to Pennsylvania, which means the results may not be generalizable to other states. Although unlikely, there is a possibility that emergency hospitals in Pennsylvania are different in quality compared to those in other states, which can influence the outcomes. Second, we assumed that all emergency hospitals in Pennsylvania, within and between counties, provide the same level of service. Any significant variation in service qualities across counties could affect the survival rate of victims of crime and, consequently, the homicide rate. Third, while county-level analysis provides useful insights into the relationship between emergency hospital services and homicide outcomes, it may overlook smaller-scale social dynamics, such as localized interpersonal conflicts that often influence violent crime in neighborhoods. Additionally, county-level data limits our ability to account for specific parameters of emergency response, such as response time, distance to the nearest emergency department, and differences in victim outcomes (e.g., Dead on Arrival, dead en route, or passed away in hospital). Future research could benefit from exploring smaller geographic units and incorporating micro-level data to better capture these localized patterns and nuances in emergency medical service effectiveness. Another limitation would pertain to the international variation in how a case is defined as homicide. If a certain jurisdiction or country includes attempted homicide or involuntary manslaughter, such as killing a random victim by car accident, it would generate relatively high counts of homicide than most jurisdictions in the USA as well as Pennsylvania. If victim's survival does not matter to the definition of homicide, the proximity to medical services would not contribute to a lower homicide rate in that particular jurisdiction. Finally, it is possible that hospitals near county borders treated victims from adjacent counties, which could threaten the measurement validity of the homicide rate by potentially counting criminal fatalities that originated in one county but were treated in another.

Despite these limitations, this study offers important implications for both future empirical research and health policy. First, our findings suggest that emergency medical service availability should be considered when examining homicide rates in cross-jurisdictional studies, as it may help reduce homicide lethality by enhancing access to timely trauma care. Second, regional governments may benefit from evaluating emergency hospital distribution as part of their efforts to address homicide rates. While building additional emergency hospitals may not directly address the root causes of violence or homicide, improving access to emergency medical services in high-crime areas can enhance survival rates for victims of violent incidents. Governments and health policymakers may consider optimizing the placement and accessibility of emergency medical services, which may involve enhancing trauma care, reducing emergency response times, and developing partnerships between hospitals and urgent care facilities. Such improvements could produce immediate benefits for public health and safety by reducing mortality in regions with high rates of violent crime.

## CRediT authorship contribution statement

**Mingean Park:** Writing – original draft, Methodology, Formal analysis, Conceptualization. **Sujeong Park:** Writing – review & editing, Formal analysis, Conceptualization. **Youngeun Lee:** Writing – original draft, Resources, Methodology, Data curation. **Jonathan Lee:** Writing – review & editing, Methodology.

## Ethical statement

The authors acknowledge that

• the work described has not been published previously except in the form of a preprint, an abstract, a published lecture, academic thesis or registered report.
• the article is not under consideration for publication elsewhere.
• the article's publication is approved by all authors and tacitly or explicitly by the responsible authorities where the work was carried out.
• if accepted, the article will not be published elsewhere in the same form, in English or in any other language, including electronically without the written consent of the copyright-holder.

## Disclosure statement

The authors report there are no competing interests to declare.

## Data Availability

Data will be made available on request.
